# Transdiaphragmatic Rupture of Hepatic Hydatid Cyst With Pleural Effusion and Daughter Cysts: A Case Report and Literature Review

**DOI:** 10.1002/ccr3.71970

**Published:** 2026-02-02

**Authors:** Zahra Sadin, Manochehr Aghajanzadeh, Mohammadreza Sadin, Mohaya Farzin

**Affiliations:** ^1^ Department of Thoracic Surgery Guilan University of Medical Sciences Rasht Iran; ^2^ Department of Pulmonology Guilan University of Medical Sciences Rasht Iran; ^3^ Department of Internal Medicine, Inflammatory Lung Diseases Research Center, Razi Hospital, School of Medicine Guilan University of Medical Sciences Rasht Iran

**Keywords:** daughter cysts, diaphragmatic rupture, echinococcosis, hydatid cyst, pleural effusion

## Abstract

Trans‐diaphragmatic rupture of a hepatic hydatid cyst, can manifesting as a pleural effusion with daughter cysts, which could be an uncommon but serious complication. Prompt diagnosis through imaging and timely surgical intervention are critical to prevent life‐threatening outcomes in endemic areas.

## 1 Introduction

Hydatid cysts, caused by Echinococcus granulosus infection, are a significant public health challenge in endemic regions, with 2–3 million cases globally [1, 2]. The liver is affected in 60%–75% of cases, followed by the lungs in 15%–20% [2]. While rupture occurs in 30%–50% of complex cases, transdiaphragmatic rupture into the pleural cavity is rare (approximately 3%), leading to pleural effusion, hepatopleural fistula, or empyema with daughter cysts [2, 3, 4, 5]. Historical data from a Greek hospital (1914–1961) documented 1198 hepatic hydatid cyst cases, with only 0.77% progressing to pleural rupture and fistula formation [5]. Hepatopleural fistulas may present with acute symptoms like chest pain, dyspnea, or cyanosis [6]. Diagnosis is confirmed by imaging, preferably CT, which reveals diaphragmatic fistulas, pleural effusion, and cystic lesions consistent with daughter cysts [7]. Surgery, including pericystectomy or thoracotomy with diaphragm repair, is the mainstay of treatment, but it needs careful cyst evacuation to prevent spillage and recurrence [1]. Adjuvant albendazole therapy, administered pre‐ and post‐operatively, reduces recurrence risk [8]. This report presents a rare case of transdiaphragmatic rupture of a hepatic hydatid cyst manifesting as pleural effusion with daughter cysts. It emphasizes the need for prompt diagnosis and urgent surgical intervention in endemic regions.

## 2 Case History

A 54‐year‐old woman presented to our emergency department with right‐sided chest pain, shortness of breath, and tachypnea. On arrival, her respiratory rate was 22 breaths per minute, and auscultation revealed diminished breath sounds over the right hemithorax. She lived in a rural village where she was in close contact with livestock, including sheep and cattle.

**FIGURE 1:**
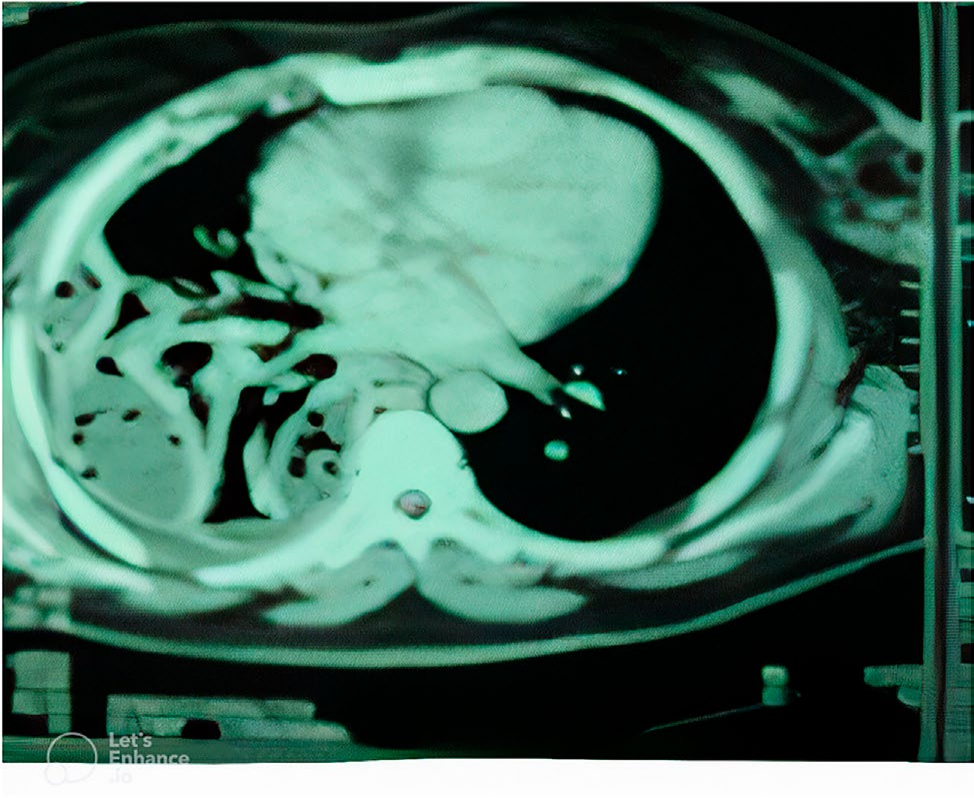
CT‐scan shows pleural effusion with air space.

**FIGURE 2, 3, 4:**
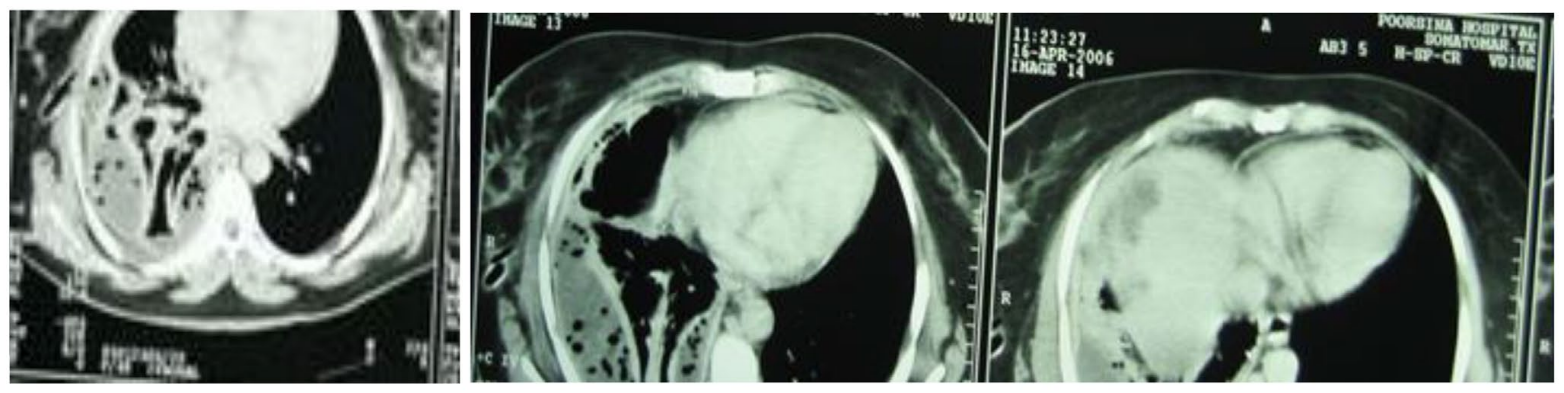
CT‐scan shows pleural effusion with air space and chest tube with collapse of lung.

On abdominal examination, we noted two well‐healed right subcostal surgical scars, which were consistent with a history of prior operations for hepatic hydatid cysts. A chest X‐ray showed a right‐sided pleural effusion. It was further characterized by a chest CT scan, which revealed a significant pleural fluid collection, partial collapse of the right lower lobe, and multiple internal air lucencies within the effusion (Figures 1, 2, and 3).

An urgent thoracentesis was performed, followed by the insertion of a chest tube. Analysis of the pleural fluid revealed a low glucose concentration, acidic pH, elevated white blood cell count (~1000 cells/μL), and markedly increased lactate dehydrogenase (LDH: 825 mg/L), all suggestive of an empyema.

**FIGURE 5, 6, 7, 8:**
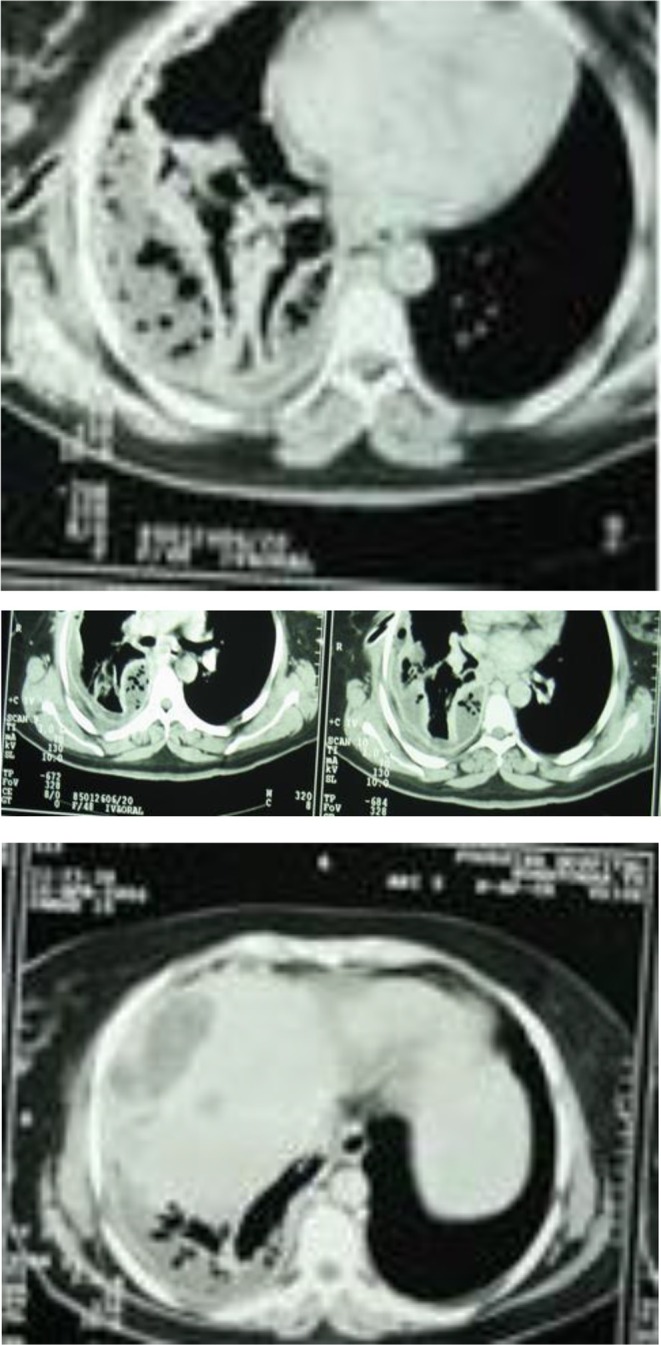
CT‐scan of abdomen, which shows cystic lesions in the liver with pleural effusion.

Further imaging with an abdominal CT scan identified a cystic lesion on the dome of the right hepatic lobe (Figures 4 and 5), which raised suspicion for a recurrent hydatid cyst. Despite initial drainage, a follow‐up chest X‐ray on day five showed persistent right lung collapse. A flexible bronchoscopy was performed to rule out endobronchial obstruction. It revealed external compression of the right lower lobe without evidence of an intraluminal lesion.

Taken together, these findings supported a diagnosis of intrathoracic rupture of a hepatic hydatid cyst into the pleural space. The patient underwent a right posterolateral thoracotomy. Intraoperative exploration revealed multiple fragments of laminated membranes, daughter cysts, and hydatid fluid, which was consistent with hydatid disease (Figures 6–8).

Following complete evacuation of the pleural cavity, which contained hydatid fluid, daughter cysts, and laminated membranes, decortication and pneumonolysis were performed to allow full lung re‐expansion. In the course of the procedure, a 4 cm fistulous tract was identified, which extended from the hepatic dome through the diaphragm into the pleural space. A phrenotomy was carried out to access the hepatic cyst, which was subsequently evacuated. A 24 French Foley catheter was placed into the residual hepatic cavity for continued drainage.

**FIGURE 9, 10, 11:**
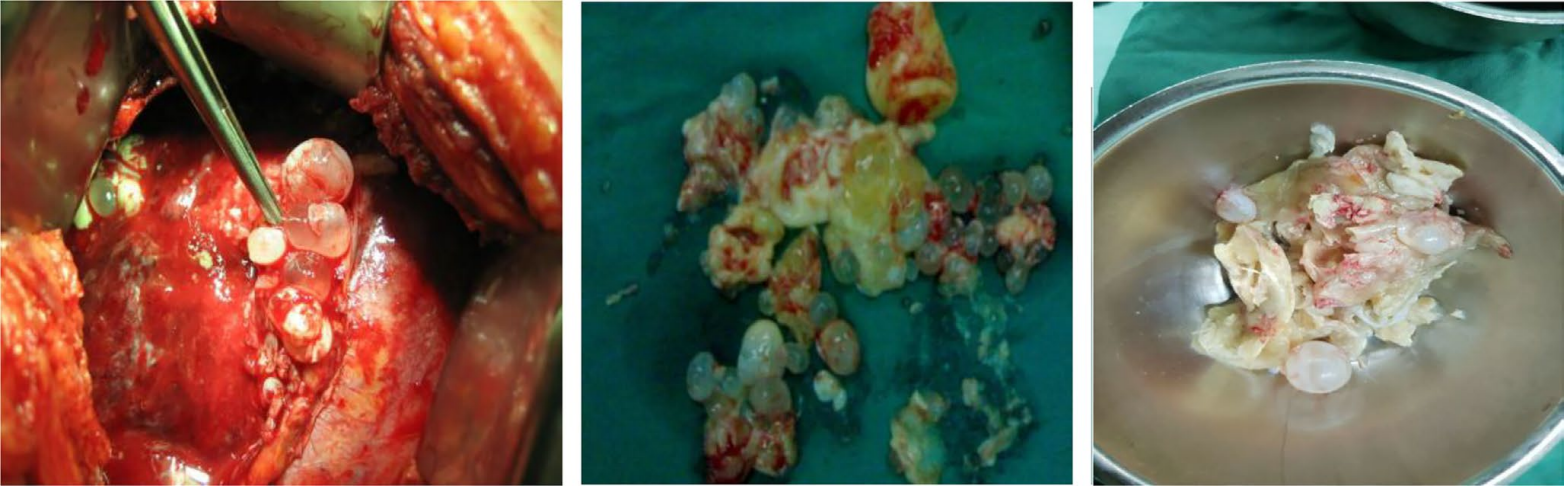
Multiple daughter cysts in the pleural space and laminated membranes.

Intraoperative findings confirmed the presence of multiple daughter cysts, laminated membranes, and a 4 cm diaphragmatic fistula (Figures 9–11).

The patient was transferred to the intensive care unit for close monitoring and supportive care over the next 2 days. Albendazole therapy was initiated postoperatively. Her recovery was uneventful, and she was discharged on postoperative day six in good general condition.

## 3 Conclusion

Transdiaphragmatic rupture of a hepatic hydatid cyst, is a rare complication, which has been seen in 1%–3% of cases. it presents with pleural effusion and daughter cysts, as observed in this patient with a 4 cm fistulous tract. Clinicians in endemic regions should prioritize hydatid disease in the differential diagnosis of unexplained pleural effusion. They can rely on CT imaging for prompt diagnosis. Urgent surgical intervention, combined with albendazole therapy, is critical to prevent complications like empyema. Future studies should explore minimally invasive approaches to improve outcomes.

## 4 Discussion

Transdiaphragmatic rupture of a hepatic hydatid cyst, as observed in this case, is an uncommon complication, which only occurs in 1%–3% of cases. It can result in pleural effusion and hepatopleural fistula formation [2, 5]. Historical data from a Greek hospital (1914–1961) reported only 0.77% of 1198 hepatic hydatid cyst cases that progress to pleural rupture. It underscores its rarity [5]. Previous reports describe similar presentations with pleural effusion and daughter cysts [1, 4, 7], but our case is distinguished by a 4 cm fistulous tract and extensive pleural involvement, which necessitates open thoracotomy. Recent literature highlight minimally invasive approaches, such as ERCP or thoracoscopy, as alternatives to traditional surgery in select cases [6, 8].

The diagnostic challenge is due to nonspecific symptoms like chest pain and dyspnea, which may mimic empyema or malignancy [7, 9]. CT imaging is essential to timely diagnosis, because it can reveal the diaphragmatic fistulas and daughter cysts [7]. Surgical intervention, including thoracotomy, pericystectomy, and diaphragm repair, remains the primary approach. It is crucial to exercise caution to prevent intraoperative spillage and reduce recurrence and anaphylaxis risk [1, 4, 8]. Albendazole therapy (10–15 mg/kg/day), administered for 2–3 months before and after surgery, plays a vital role in sterilizing hydatid cysts and reducing the likelihood of recurrence [6, 8, 10].

In regions where hydatid disease is prevalent, clinicians should prioritize including it in the differential diagnosis for patients presenting with unexplained pleural effusion [1, 2, 7]. Prompt CT imaging and urgent surgical intervention are critical to prevent complications such as empyema or pneumothorax [1, 7]. In select cases, minimally invasive techniques like ERCP or PAIR (puncture, aspiration, injection, re‐aspiration) offer promising alternatives [6, 10]. However, this case is limited by its single‐patient nature and the absence of serological testing due to low sensitivity. Future studies should explore minimally invasive approaches and standardized protocols to optimize management of transdiaphragmatic rupture [6, 10].

## Author Contributions

**Zahra Sadin:** conceptualization, data curation, formal analysis, funding acquisition, investigation, methodology, project administration, resources, software, supervision, validation, visualization, writing – original draft, writing – review and editing. **Manochehr Aghajanzadeh:** conceptualization, data curation, formal analysis, funding acquisition, investigation, methodology, project administration, resources, software, supervision, validation, visualization. **Mohammadreza Sadin:** conceptualization, data curation, formal analysis, funding acquisition, investigation, methodology, project administration, resources, software, supervision, validation, visualization, writing – original draft, writing – review and editing. **Mohaya Farzin:** conceptualization, data curation, formal analysis, funding acquisition, investigation, methodology, resources, software.

## Funding

The authors have nothing to report.

## Ethics Statement

Ethical approval was not required for this case report. However, all procedures performed were in accordance with institutional and/or national ethical standards.

## Consent

Written informed consent was obtained from the patient for publication of this case report and accompanying images.

## Conflicts of Interest

The authors declare no conflicts of interest.

## Data Availability

Data sharing is not applicable to this article as no datasets were generated or analyzed during the preparation of this case report.
